# Ultrafast Optical Kerr Effect Spectroscopy Reveals the Vibrational Fingerprint of Acetate–Water Hydrogen Bonds

**DOI:** 10.1021/acsomega.5c09879

**Published:** 2025-12-01

**Authors:** Yousaf Shah, Stephen R. Meech, Ismael A. Heisler

**Affiliations:** † Instituto de Física, Universidade Federal do Rio Grande do Sul - UFRGS, Avenida Bento Gonçalves, 9500 Porto Alegre, Brazil; ‡ School of Chemistry, Norwich Research Park, 6106 University of East Anglia, Norwich NR4 7TJ, U.K.

## Abstract

Time-resolved optical Kerr effect (OKE) spectroscopy was employed to investigate the low-frequency vibrational dynamics of aqueous acetate solutions. While the isotropic OKE spectrum of neat water is broad and featureless, acetate solutions display a distinct band near 200 cm^–1^. This feature increases systematically with acetate concentration, is absent in methyl acetate, and shows negligible dependence on the countercation, establishing it as the vibrational fingerprint of acetate–water hydrogen bonds. Comparison with hydroxide solutions demonstrates that the band is spectrally distinct from other anion–water vibrations. Quantum-chemical calculations further support the assignment, reproducing polarized vibrational modes in the same frequency region. Together, these results resolve long-standing ambiguities in the interpretation of acetate hydration and highlight the power of ultrafast OKE spectroscopy to isolate solute-specific hydrogen-bond vibrations in aqueous solutions. Beyond spectroscopy, these findings have implications for understanding electrolyte behavior in energy storage systems (e.g., lithium-ion batteries) and biological buffering processes.

## Graphical abstract

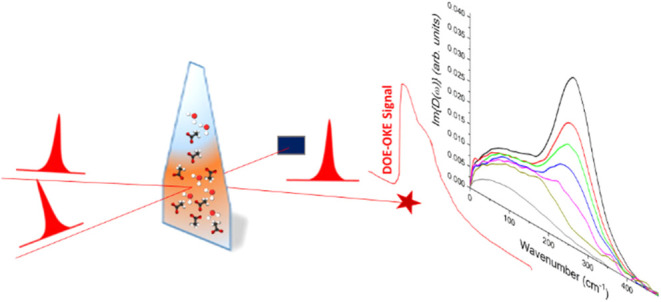

## 1 Introduction

The hydration of small carboxylate ions such as acetate (CH_3_COO^–^) lies at the heart of numerous chemical, biological, and environmental processes.^1,2^ As a simple, prototypical monovalent anion, acetate serves as a model system for understanding the broader principles of ion hydration, solvation dynamics, and solute–solvent interactions.^3–5^ Its role as a conjugate base of acetic acid also makes it a key component in biological buffering systems, enzymatic regulation, and acid–base equilibria in aqueous environments.^6^ Despite its apparent simplicity, the hydration structure and dynamics of acetate remain an active area of investigation due to the complex interplay between electrostatic forces, hydrogen bonding, and thermal fluctuations in the aqueous phase.^2,7–12^

Historically, studies of ion hydration focused on hydration enthalpies, activity coefficients, and scattering methods to infer solvation structure. While valuable, such ensemble-averaged techniques could not capture the ultrafast fluctuations of hydrogen-bond networks or transient hydration structures.^13–15^ In the latter half of the century, nuclear magnetic resonance (NMR) and infrared (IR) spectroscopy provided new insights into the average structure of hydration shells,^16–18^ while X-ray and neutron scattering enabled the determination of radial distribution functions around solvated ions.^19–21^ However, these ensemble-averaged or time-integrated techniques often failed to resolve the dynamic nature of hydration — particularly the ultrafast fluctuations of hydrogen-bond networks and the transient structures formed in solution.^16,22,23^

More recently, the application of ultrafast spectroscopic methods, such as time-resolved infrared and optical Kerr effect (OKE) spectroscopy, has opened a window into the picosecond and subpicosecond dynamics of ion hydration.^24–27^ These techniques reveal how water molecules respond collectively to the presence of an ion and how energy redistributes through coupled vibrational modes.^23,24,26^ For acetate, such studies have shown that the ion does not behave as an inert electrostatic center; rather, it actively participates in shaping the local hydrogen-bond network and modulates the low-frequency vibrational landscape of the surrounding solvent.^28–30^ In particular, the bending and twisting motions of the hydration shell — which occur in the low-wavenumber (<300 cm^–1^) regime — provide direct evidence of strong solute–solvent coupling and ion-specific dynamical signatures.^30–34^

Understanding the hydration of acetate ions is therefore more than a niche pursuit: it informs fundamental theories of solvation, aids in the refinement of molecular dynamics force fields, and supports the development of accurate predictive models for aqueous systems.^1,35–37^ In this work, we employ time-resolved transient grating OKE spectroscopy to investigate the low-frequency vibrational dynamics of aqueous acetate solutions.^26,38^ By analyzing the isotropic response, we aim to resolve the distinct vibrational signatures of ion–solvent interactions separately from the otherwise dominant solvent–solvent signals and thus, characterize the relaxation pathways within the hydration shell. These observations contribute to a more complete picture of how carboxylate ions modulate water structure and dynamics at ultrafast time scales.^26,39,40^ Despite decades of Raman, IR, and dielectric studies, the specific vibrational fingerprint of acetate hydration has remained unresolved, creating ambiguity in theoretical and experimental interpretations.^41–44^

## 2 Experimental Methods

Water, salts, and hydroxides used in this study were of analytical grade (Sigma-Aldrich). The prepared solutions were filtered through 0.2 μm micropore filters to eliminate particulate matter before being loaded into fused silica cuvettes with a 2 mm path length. This cuvette length was chosen to minimize signal contributions from the cell walls, ensuring that the optical beams intersected solely within the liquid phase, which is critical for detecting the relatively weak isotropic response.

The experimental setup closely followed configurations reported in previous studies.^40^ In brief, isotropic time-resolved OKE measurements were performed using ultrashort pulses generated by a commercial Kerr-lens mode-locked Ti:sapphire laser (Micra 10, Coherent). The laser output featured a central wavelength of approximately 800 nm, an 80 nm bandwidth, and an average power of 800 mW. A pair of fused silica prisms was used both to correct temporal broadening from the oscillator output and to precompensate for dispersion introduced by the optical elements in the system. Autocorrelation measurements at the sample position, using a 50 μm BBO crystal, confirmed typical pulse durations of 20 fs.

To access all components of the third-order nonlinear susceptibility, we employed a diffractive-optics-based transient grating spectrometer.^40,45^ Our primary interest was in the isotropic response, which was isolated by setting the probe polarization parallel to the analyzing polarizer and at 54.7° relative to the pump polarization—commonly referred to as the magic angle. The third-order nonlinear signal generated in the sample was mixed with a fourth beam, known as the local oscillator (LO), derived from the diffractive optics element, enabling fully heterodyne-detected measurements. The relative phase between the LO and the signal field was finely adjusted to 90° using thin glass plates, allowing selective detection of the birefringent contribution.^40^

The measured signal, denoted as *S*(*t*), represents a convolution between the sample’s polarizability response function, *R*(*t*), and the instrument response function, *G* ^(2)^(*t*), the second-order autocorrelation of the laser pulses, so the convolution can be written as^46^

1 $$S\left(t\right)=R{\left(t\right)}^{*}G^{\left(2\right)}\left(t\right)$$

The dynamics captured by time-resolved OKE can be analyzed in both the time and frequency domains. A frequency-domain representation is obtained via the Fourier transform deconvolution:^46^

2 $$\frac{\mathrm{FT}\left[S\left(t\right)\right]}{\mathrm{FT}\left[G^{\left(2\right)}\left(t\right)\right]}=D\left(\omega \right)$$

where *FT* stands for Fourier transform. The imaginary part of *D*(ω), i. e., Im {*D*(ω)}, reflects only the nuclear contribution to the polarizability anisotropy response, and is referred to as the Raman spectral density (RSD). Further experimental and theoretical details regarding time-resolved OKE spectroscopy can be found in the literature.^46,47^

## 3 Results and Discussions

OKE is a third-order nonlinear optical method that measures the transient birefringence induced by polarizability fluctuations.^47^ In practice, an ultrashort pump pulse induces a transient change in the molecular polarizability, which is subsequently detected by a time-delayed probe pulse. The resulting signal can be decomposed into anisotropic and isotropic components, corresponding respectively to orientational correlation functions and to density or interaction-induced fluctuations of polarizability.^39^ This symmetry separation ensures that the isotropic channel reports exclusively on interaction-induced, collective fluctuations, while the anisotropic channel is dominated by reorientational dynamics. Importantly, OKE spectroscopy provides access to low-frequency intermolecular vibrations (<300 cm^–1^), where collective hydrogen-bond and solute–solvent modes dominate, and thus reveals aspects of liquid-state dynamics inaccessible to linear spectroscopies.^26,38,47,48^ Unlike higher-frequency intramolecular vibrations, these low-frequency modes report directly on the collective behavior of solvent cages, hydrogen-bond networks, and solute–solvent complexes.^49–51^

In aqueous systems, the isotropic OKE response is of particular interest because it is not directly sensitive to single-molecule reorientation, which dominates the anisotropic response.^26^ Instead, the isotropic signal arises mainly from interaction-induced polarizability terms, including higher-order dipole–induced–dipole couplings first analyzed by Mazzacurati and co-workers.^52,53^ As such, it highlights the collective vibrational modes associated with transient solute–solvent complexes and hydrogen-bond stretching or bending. This selectivity makes the isotropic channel especially valuable for identifying solute-induced vibrational features that are otherwise masked by the strong librational background in Raman spectra or hidden in dielectric relaxation experiments.^49^

For neat water, the isotropic OKE response is extremely weak and decays rapidly, following an approximately single-exponential profile with a time constant of ∼ 50 fs.^26^ The corresponding frequency-domain spectrum is broad and featureless, as shown in the Supporting Information (Figure S1), and contains no distinct vibrational bands assignable to the hydrogen-bond network. Such features are present in the anisotropic response, as we and others have shown previously, but not in the isotropic channel.^26,39,54,55^ This absence establishes the isotropic spectrum of water as a clean and reliable baseline against which new solute-induced features can be recognized. To confirm this baseline, both isotropic and anisotropic OKE spectra of pure water were included for comparison in the Supporting Information, illustrating the previously studied distinction between structureless isotropic and more complex anisotropic responses.

The introduction of acetate ions induces striking and reproducible changes in the isotropic OKE spectra. At 6 M KOAc (potassium acetate) (Figure 1), the frequency-domain spectrum displays a well-defined band centered at ∼200 cm^–1^, which is absent in pure water and therefore represents a solute-induced vibrational mode. Two additional features appear at higher frequencies, near 650 and 925 cm^–1^, corresponding to the acetate δCO_2_ bending and νC–C stretching vibrations.^43^

**Figure 1:**
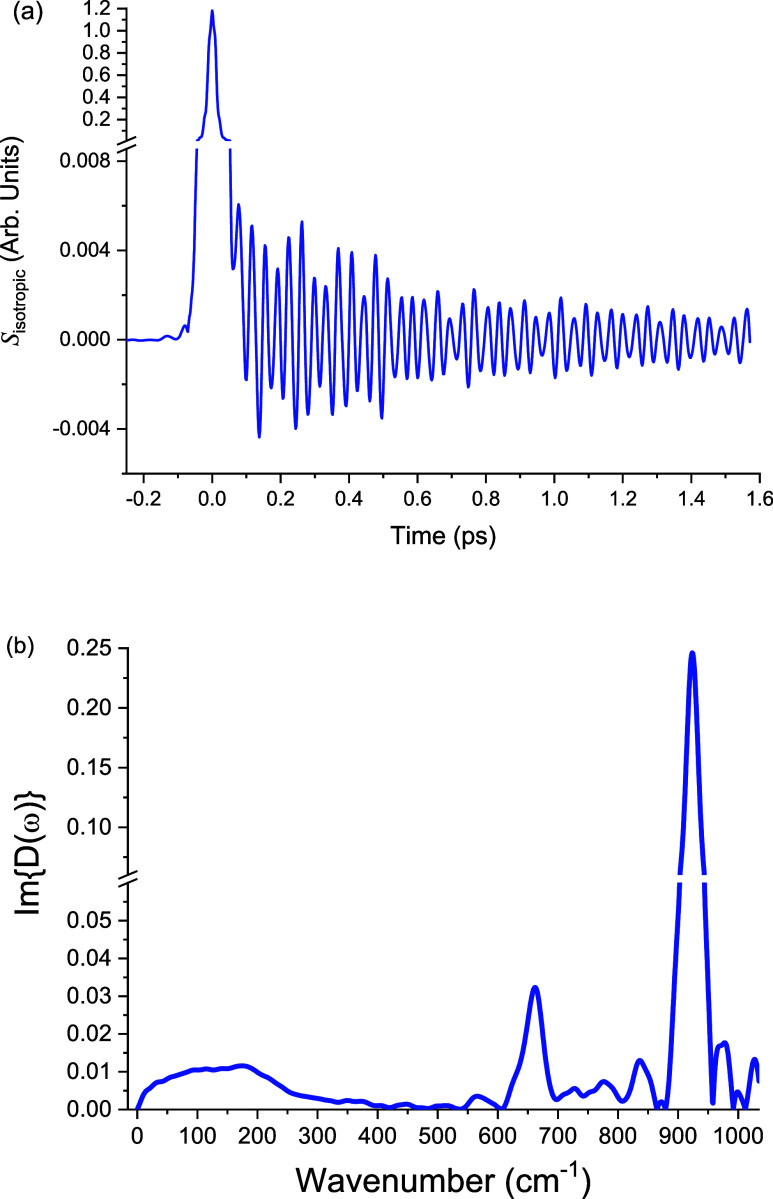
Isotropic OKE response of a 6 M KOAc solution. (a) Time-domain isotropic OKE signal showing acetate vibrations. (b) Corresponding frequency-domain spectrum obtained from Fourier transformation of the time-domain data. In addition to the low-frequency acetate–water hydrogen bond vibration at ∼200 cm^–1^, two higher frequency modes are also observed, near 650 and 925 cm^–1^, which are assigned to the acetate intramolecular δCO_2_ bending and νC–C stretching vibrations, respectively.

As these are established intramolecular modes of the acetate anion, they are not relevant to the present discussion and will not be considered further. The ∼200 cm^–1^ band, by contrast, falls within the spectral region typically associated with hydrogen-bond stretching motions and is consistent with vibrations involving acetate–water hydrogen bonds. Its observation in the isotropic response underscores its polarized nature, reflecting collective fluctuations of acetate and surrounding water molecules that modulate the isotropic polarizability tensor.

The assignment is reinforced by systematic concentration studies, which rule out artifacts and demonstrate reproducibility. Figure 2 shows isotropic OKE spectra of KOAc solutions across a series of concentrations. The amplitude of the ∼200 cm^–1^ band increases monotonically with acetate concentration, while the frequency and bandwidth remain essentially unchanged.

**Figure 2:**
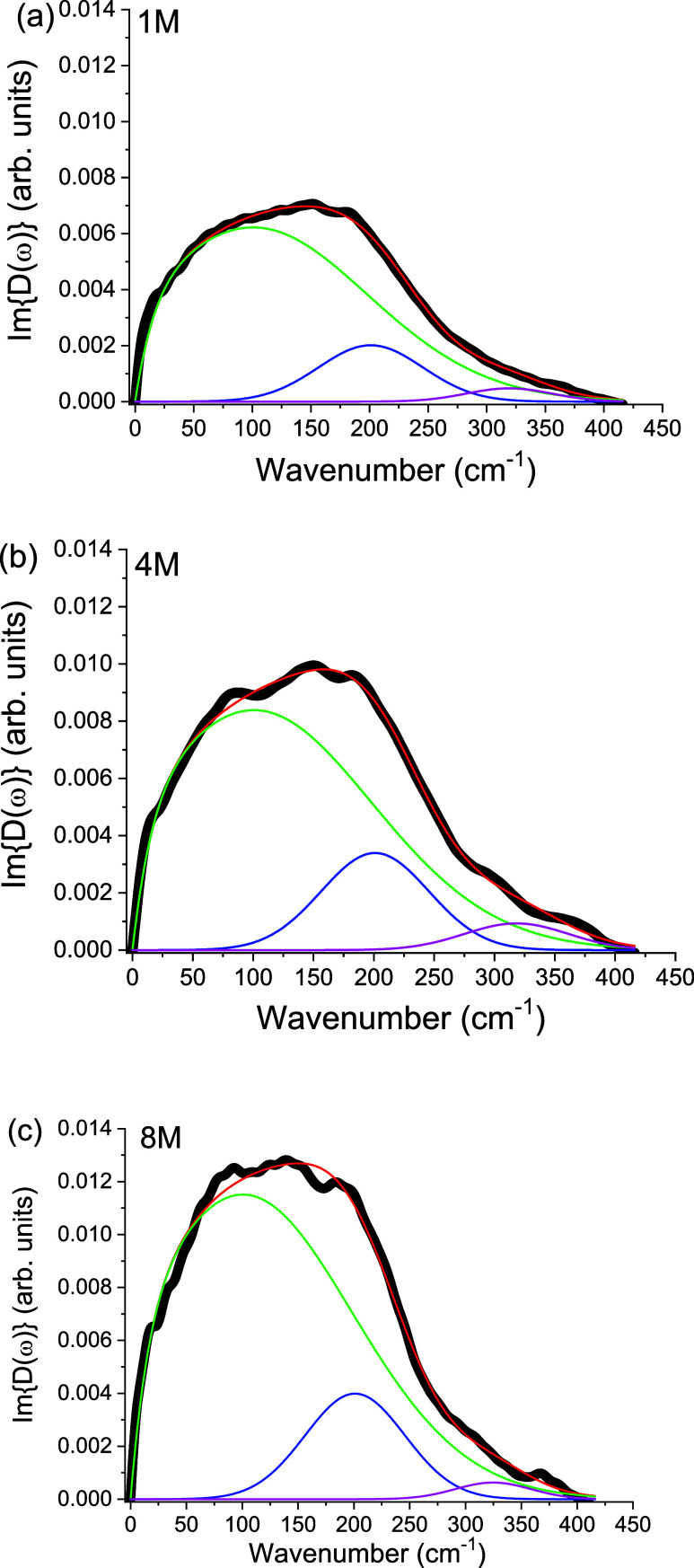
Concentration dependence of the isotropic OKE spectra of the KOAc solutions corresponding to (a) 1M, (b) 4 M and (c) 8M, with fitted components. Experimental isotropic OKE spectra (symbols) and model fits (solid lines) for aqueous acetate solutions. Individual Gaussian components and the Bucaro–Litovitz baseline are indicated.

This scaling behavior demonstrates that the vibrational mode intensity is directly proportional to the number of acetate–water hydrogen bonds in solution. In other words, the band reflects a local solute–solvent vibrational fingerprint that accumulates linearly with the number of acetate ions present. Quantitative fitting of the spectra provides a more detailed measure of this scaling. Following the procedure used in our earlier studies of hydrated ions, the low-frequency region of the isotropic spectrum was fitted with a Bucaro–Litovitz (BL) relaxation function combined with an asymmetric Gaussian function, while the higher-frequency region was reproduced using one or two additional Gaussian functions.^9^ This combination reliably reproduces the spectra across all concentrations. The robustness of the fits was tested by varying the BL relaxation parameters within the range that preserved acceptable residuals in the 0–120 cm^–1^ region. Within this range, the integrated area and centroid of the ∼200 cm^–1^ Gaussian changed by less than 5% and 3 cm^–1^, respectively. Weak undulations in the fitted envelope at high concentrations are consistent with minor baseline modulations rather than the emergence of an additional vibrational mode.

To minimize ambiguity in band decomposition, the acetate–water bandwidth and asymmetry were globally constrained across concentrations, with only amplitude and a small centroid refinement varying. The BL function was employed to model the interaction-induced low-frequency envelope in OKE, as commonly used for collective relaxation backgrounds in liquids. Under these constraints, the area of the ∼200 cm^–1^ band scales nearly linearly with acetate concentration (Figure 2), (as will be quantitatively shown next), consistent with a local acetate–water H-bond motif whose population increases with solute concentration. The invariance across cations (Figure S2) and the absence in methyl acetate (Figure S3) further support a solute–solvent H-bond origin rather than cation-dependent or bulk-water artifacts. Although partial ion pairing and first-shell depletion are expected above ∼7 M, the OKE response primarily reflects interaction-induced polarizability within local acetate–water motifs. Even when contact ion pairs form, a significant fraction of acetate anions remains hydrogen-bonded to at least one water molecule, producing a similar collective coordinate. Consequently, the integrated amplitude of the ∼200 cm^–1^ band remains approximately proportional to the number of such motifs, yielding an apparently linear trend up to 8 M.

Figure 3a shows the integrated area of the fitted acetate-induced band around ∼200 cm^–1^ as a function of concentration, already including data obtained by mixtures with a given set of hydroxides which will be discussed below.

**Figure 3:**
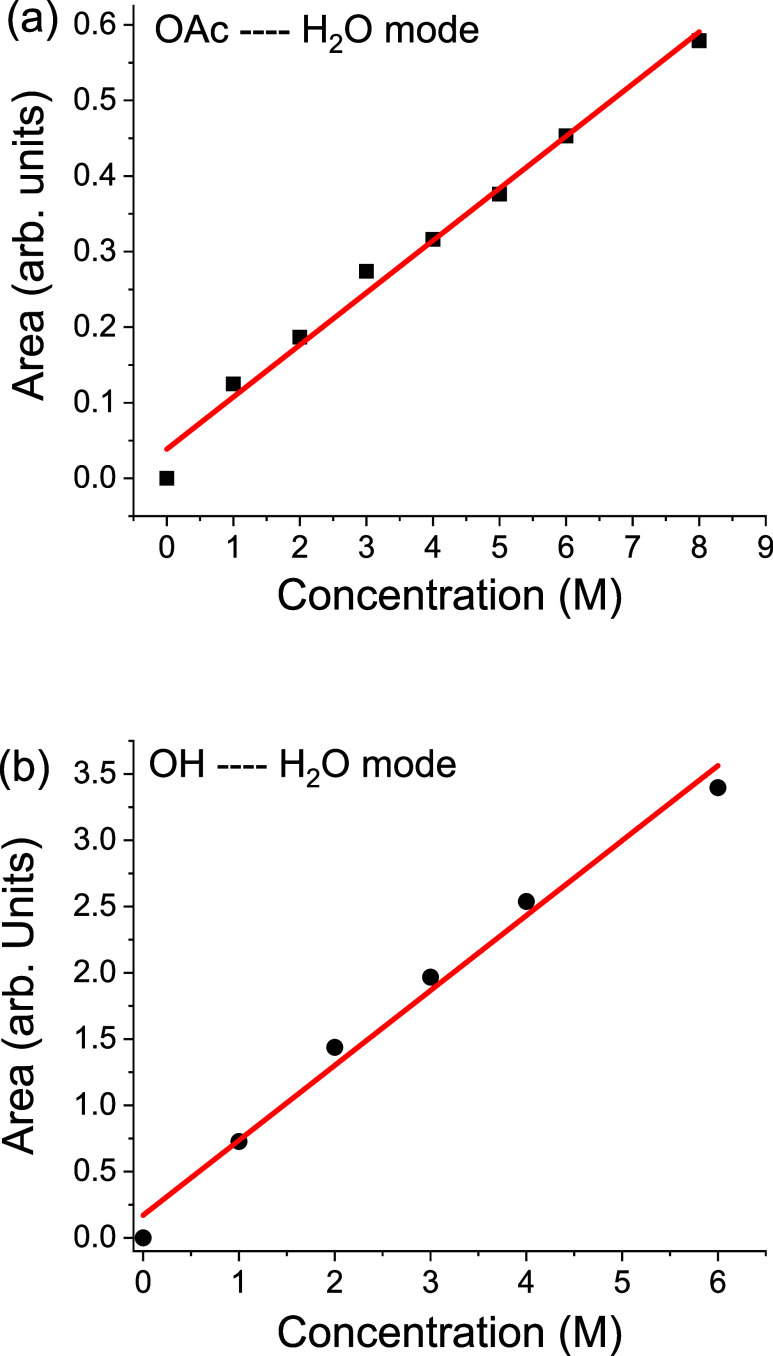
(a) Integrated area of the acetate–water band (∼200 cm^–1^) as a function of acetate concentration (1–8 M). (b) Integrated area of the hydroxide–water band (260–280 cm^–1^) as a function of hydroxide concentration for comparison. Lines are guides to the eye. Axes correspond to integrated intensity (arbitrary units) versus solute molarity (M).

The nearly linear trend provides strong evidence that the observed mode is intrinsic to acetate hydration rather than arising from extrinsic effects such as aggregation, impurities, or instrumental artifacts. This behavior rules out artifacts such as beam drift or baseline instabilities and indicates a true solute–solvent vibrational origin.

Additional comparisons further confirm the solute-specific nature of this vibrational feature. Figure 4 summarizes the isotropic response of mixtures of KOAc and KOH as the anion concentration is varied while keeping [K^+^] constant at 6 M.

**Figure 4:**
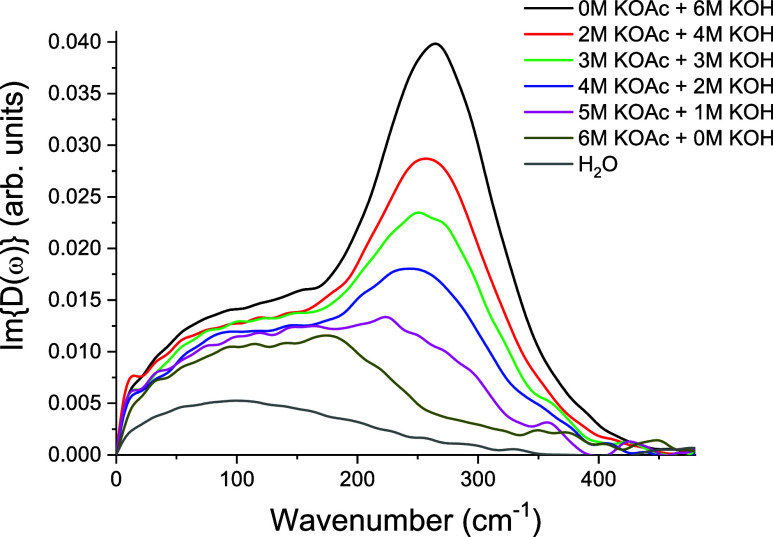
Comparison of isotropic OKE spectra for KOAc and KOH solutions at fixed [K^+^] = 6 M, illustrating the distinct acetate (∼200 cm^–1^) and hydroxide (∼260–280 cm^–1^) features and their systematic evolution as the anion concentration varies.

The data clearly show that the acetate band near 200 cm^–1^ and a hydroxide band near 260–280 cm^–1^ emerge and evolve systematically with varying anion concentration, reflecting their distinct hydration signatures. Furthermore, Figure 5 highlights the clear spectral separation between the two species. Whereas KOAc displays only the ∼200 cm^–1^ band (Figure 5a), KOH exhibits a higher-frequency feature (260–280 cm^–1^, Figure 5d) characteristic of hydroxide–water hydrogen-bond stretching.^27^ Furthermore, the integrated area of the ∼280 cm^–1^ band (Figure 3b), associated with the hydroxide solute–solvent H-bond scales linearly with hydroxide concentration, confirming the origin of this spectral band.

**Figure 5:**
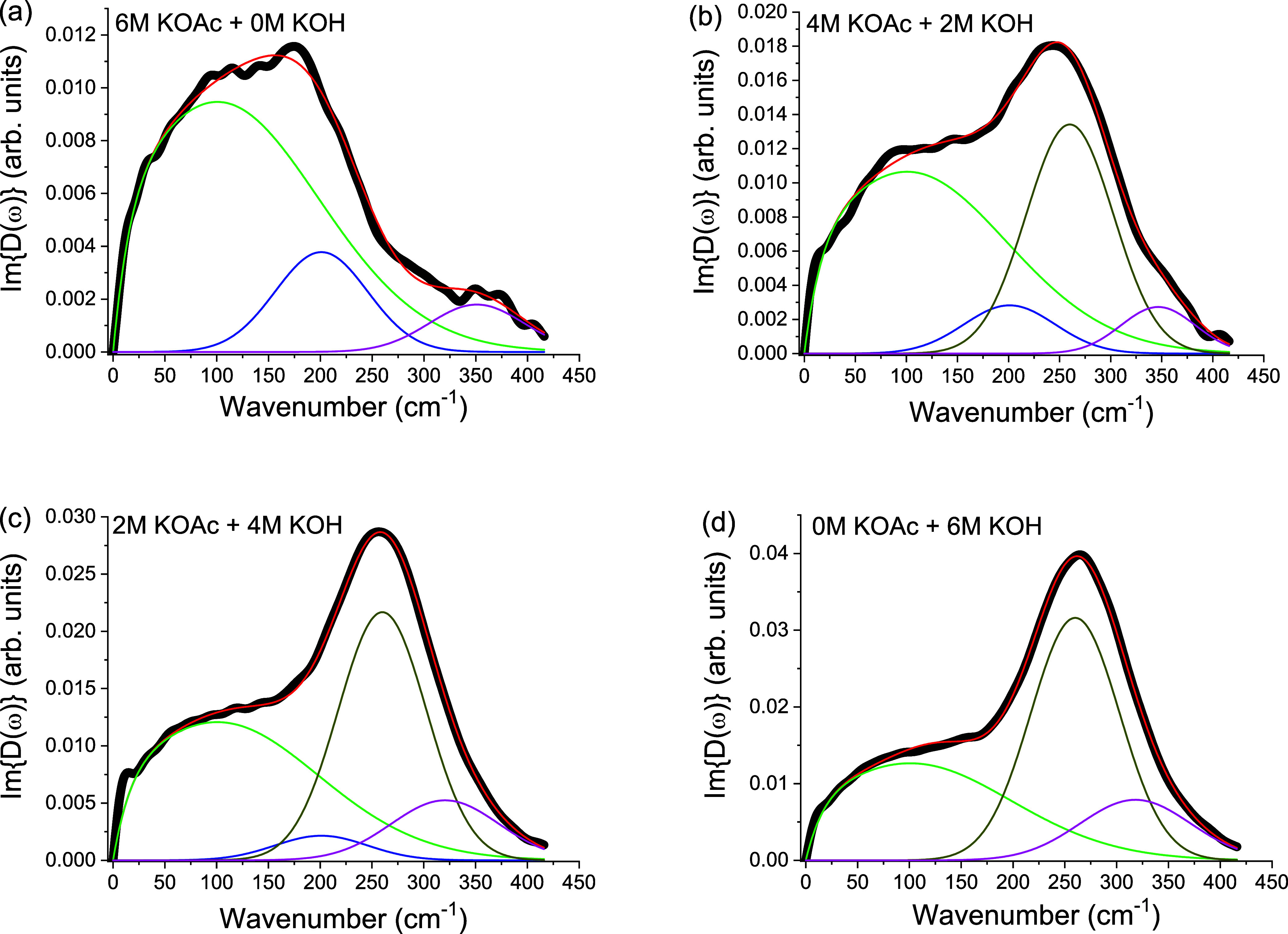
Isotropic OKE spectra of KOAc and KOH solutions at fixed [K^+^] = 6 M, with the following concentration combinations: (a) 6 M KOAc + 0 M KOH, (b) 4 M KOAc + 2 M KOH, (c) 2 M KOAc + 4 M KOH and (d) 0 M KOAc + 6 M KOH. This shows the clear spectral separation between the acetate (∼200 cm^–1^) and hydroxide (∼260–280 cm^–1^) bands, a distinction which underscores the ion-specific nature of the vibrational fingerprints.

This contrast underscores the ion-specific vibrational fingerprints revealed by isotropic OKE spectroscopy.^26^ In these mixtures, the concentrations of KOAc and KOH were varied simultaneously while maintaining a constant total [K^+^] = 6 M. This design allowed us to compare ion-specific vibrational fingerprints under identical cation environments. We recognize that holding one species constant while varying the other would further isolate anion-specific contributions, and such a configuration will be implemented in future measurements to decouple potential cross-effects. Furthermore, the frequency difference between the acetate and hydroxide bands reflects the distinct nature of their hydrogen-bond interactions. In hydroxide solutions, the O–H···O bonds are stronger and more directional, with localized negative charge on the hydroxide oxygen leading to a stiffer collective coordinate and therefore a higher vibrational frequency (260–280 cm^–1^). In contrast, the negative charge in acetate is delocalized over the carboxylate group, and the associated hydrogen bonds exhibit greater angular and strength heterogeneity, producing a softer, lower-frequency collective stretch near 200 cm^–1^. This interpretation aligns with the ion-specific spectral separation illustrated in Figure 5.

The spectral separation demonstrates that each anion leaves a distinct vibrational fingerprint on the isotropic OKE spectrum, reflecting differences in geometry and strength of hydrogen bonding. The acetate-induced band is therefore not a generic anion–water feature but a specific marker of the acetate–water hydrogen bond. Furthermore, the band is insensitive to the identity of the countercation. Figure S2 shows that lithium, sodium, and potassium acetates all produce the same ∼200 cm^–1^ feature with negligible frequency shift. This invariance rules out assignment to cation–O ion-pair stretching vibrations or to other cation-dependent hydration effects, and firmly anchors the band to the hydration shell of the acetate anion itself.

A crucial control is provided by methyl acetate, which cannot act as a hydrogen-bond acceptor through its ester oxygen. Figure S3 compares isotropic OKE spectra of acetate salts with methyl acetate solutions. In all acetate salts, the ∼200 cm^–1^ feature is present, whereas methyl acetate solutions lack any corresponding band. This contrast demonstrates that the vibration is uniquely associated with the hydrogen-bonding function of the carboxylate group and is not attributable to hydrophobic contributions from the methyl group or to bulk-water restructuring. The methyl acetate comparison thus provides a compelling negative control, underscoring the role of the carboxylate group in generating the observed vibrational mode.

Density functional theory (DFT) calculations provide further support for our experimental observations. At the B3LYP/6–31++G­(d,p) level, we optimized acetate structures solvated by different numbers of H_2_O molecules, as illustrated in Figure S4. We also examined an optimized ion-pair structure and analyzed its normal mode Raman frequencies. It should be emphasized that these simple cluster calculations are intended to provide qualitative insight into the dominant vibrational character of acetate–water hydrogen bonds rather than a quantitative reproduction of the experimental band. The exact positions and relative intensities of the computed modes depend sensitively on the chosen level of theory—particularly the size of the basis set and inclusion of diffuse and polarization functions. Despite these limitations, the trends obtained at the B3LYP/6–31++G­(d,p) level capture the emergence of polarized low-frequency modes near 200 cm^–1^, in good accord with the observed acetate–water band. Among the various models, the configuration with four water molecules yielded the best agreement with experiment. This structure displays a well-defined hydration motif, where both carboxylate oxygens engage in hydrogen bonding, while additional solvent molecules contribute to stabilization through secondary interactions. As shown in Table SIc, the acetate–(H_2_O)_4_ cluster exhibits several low-frequency polarized modes (modes 13–15) in the 170–220 cm^–1^ region, identified by normal-mode analysis as collective O–H···O stretching vibrations of acetate–water hydrogen bonds. No empirical scaling factor was applied to the harmonic DFT frequencies reported here and in the SI; any residual mismatch with experiment is attributed to the harmonic approximation and basis-set limitations. These frequencies align closely with the experimentally observed band. The additional cluster models with one or two water molecules (Figure S4) also yield polarized low-frequency modes, but the four-water model provides the closest agreement to experiment, indicating that cooperative hydrogen bonding in a partially saturated hydration shell is required to reproduce the observed spectrum. It is important to emphasize that this four-water cluster identified in the DFT analysis should be regarded as a representative motif that captures the collective, polarized hydrogen-bond stretching motion near 200 cm^–1^, rather than as a literal solvation number applicable to all concentrations. In highly concentrated solutions (7–9 M), partial dehydration and second-shell restructuring are expected; however, a sufficient fraction of acetate anions remains hydrogen-bonded to at least one water molecule, sustaining the same local vibrational coordinate. The persistence and linear scaling of the 200 cm^–1^ band therefore reflect the population of such acetate–water motifs rather than the total hydration number. This assignment is further supported by the invariance of the band position across cations (Li^+^, Na^+^, K^+^; Figure S2), its distinction from the hydroxide band at 260–280 cm^–1^, and its absence in methyl acetate (Figure S3), all of which confirm a carboxylate–water vibrational origin. Together, the experiment and theory thus converge on the assignment of the ∼200 cm^–1^ feature to collective hydrogen-bond stretching vibrations in acetate hydration.

Furthermore, the DFT analysis is performed within the harmonic approximation and small clusters; thus, absolute frequencies can be overestimated and intensities do not map directly onto isotropic OKE strengths, which depend on interaction-induced polarizability and collective selection rules. Modes calculated in the 300–420 cm^–1^ range in cluster models are comparatively localized and do not manifest as a distinct band in the isotropic OKE spectra, which are dominated by collective, polarized fluctuations. By contrast, the acetate–water H-bond stretching near 200 cm^–1^ shows strong polarization and collective character, consistent with its clear appearance in the isotropic response. The cluster geometries analyzed here represent optimized, low-energy configurations and should therefore be regarded as qualitative snapshots of the potential energy surface. In liquid solution, thermal fluctuations will lead to a distribution of conformations and hydrogen-bond arrangements, meaning that the DFT stick spectra, presented in Figure S5 represent typical motifs rather than a population-averaged ensemble. Future computational work will extend this analysis using both classical and ab initio molecular dynamics simulations to quantify the distribution of acetate–water coordination numbers, hydrogen-bond lifetimes, and low-frequency spectral densities as a function of concentration. Such simulations will provide a population-averaged picture of the local structures and collective motions, complementing the qualitative insights obtained here from static cluster calculations.

The present results also resolve ambiguities in the existing literature. Blatz and Waldstein, in their pioneering 1968 Raman study, reported broad maxima at ∼185 and 88 cm^–1^ in concentrated acetate solutions.^41^ Given the limited spectral resolution and difficulties in subtracting the strong water background, the low-frequency maximum was almost certainly an artifact, while the ∼185 cm^–1^ band corresponds closely to the mode observed here. However, without concentration-dependent analysis, their assignment remained tentative. Buchner and Rahman, using dielectric relaxation spectroscopy, identified additional relaxation modes in NaOAc solutions at ∼8 and 0.6 GHz, attributed to slow water in hydration shells and to rare ion pairing, respectively.^56^ While valuable for characterizing reorientational and cooperative dipolar dynamics, these processes occur on time scales 3 orders of magnitude slower than the vibrational modes accessed here. The OKE-observed ∼200 cm^–1^ band therefore represents a qualitatively different regime of solute–solvent dynamics. More recently, ultrafast vibrational spectroscopies have been applied to acetate solutions. Banno et al. employed infrared pump–probe spectroscopy in D_2_O and assigned transient dynamics to local carboxylate modes, while Korotkevich and Bakker investigated acetate and terephthalate ions in water using ultrafast vibrational spectroscopy, but no distinct low-frequency band attributable to acetate–water hydrogen bonds was resolved.^29,30^ Finally, Rudolph, Fischer and Irmer combined Raman spectroscopy with DFT calculations and assigned a 189 cm^–1^ polarized band to Na–O stretching in hydrated sodium, along with a shoulder near 245 cm^–1^ attributed to restricted O–H···O translations of acetate hydration.^42,43^ Our systematic analysis refutes the Na-specific assignment: the ∼200 cm^–1^ band is invariant across cations and is absent in methyl acetate, proving that it arises from the acetate–water hydrogen bond rather than from sodium–oxygen vibrations. By integrating experimental scaling, cation variation, control molecules, and DFT, our OKE study provides the most direct and unambiguous assignment to date.

In summary, the convergence of experimental and theoretical evidence establishes the ∼200 cm^–1^ feature as the vibrational fingerprint of acetate–water hydrogen bonds. The band is absent in pure water, appears consistently in acetate solutions, scales linearly with concentration, is distinct from hydroxide–water vibrations, and is cation-insensitive. Its absence in methyl acetate confirms that it originates specifically from the hydrogen-bond acceptor function of the carboxylate group. DFT calculations reproduce polarized low-frequency modes in the same spectral region, providing theoretical support for the assignment. Together, these findings resolve long-standing ambiguities in the assignment of acetate hydration modes and demonstrate the unique capability of ultrafast isotropic OKE spectroscopy to isolate solute-specific hydrogen-bond vibrations. More broadly, they show that each anion leaves a unique vibrational imprint on the low-frequency spectrum, underscoring the potential of OKE to build a systematic spectroscopic map of ion-specific hydration dynamics.^26,27,32^

## 4 Conclusions

The present work identifies, for the first time, a well-defined vibrational mode of acetate hydration in the isotropic OKE spectrum. A band centered near 200 cm^–1^ emerges clearly in acetate solutions, absent in pure water, scales linearly with concentration, and is unaffected by cation identity. Comparison with hydroxide solutions shows that the acetate mode is spectrally distinct from other anionic hydrogen-bond vibrations, while the absence of the band in methyl acetate confirms that it arises specifically from the hydrogen-bond acceptor capacity of the carboxylate group. Density functional theory calculations reproduce low-frequency polarized vibrations in the same spectral region, providing strong theoretical support for the assignment.

Taken together, the combination of ultrafast spectroscopy, systematic controls, and quantum-chemical modeling establishes the ∼200 cm^–1^ feature as the vibrational fingerprint of acetate–water hydrogen bonds. This assignment resolves conflicting interpretations in the literature and highlights the sensitivity of OKE to solute-specific low-frequency vibrational modes. More broadly, the results underscore the potential of OKE to provide a systematic spectroscopic framework for characterizing ion-specific hydration dynamics across a wide range of biologically and technologically relevant aqueous electrolytes. Future applications of this approach may allow a systematic mapping of ion-specific vibrational fingerprints across biologically and technologically relevant electrolyte systems, including multivalent carboxylates and amino acid side chains.^57–59^

## Supplementary Material



## Notes

Additional OKE time- and frequency-domain data, control spectra of pure water, and comparative measurements for acetate salts with different counter-cations; Complementary spectra of aqueous acetates and methyl acetate are provided to highlight solute-specific vibrational features; Details of the quantum-chemical modeling are also presented, including optimized structures and tabulated normal-mode analyses with Raman activities and depolarization ratios; These materials support the assignments and interpretations discussed in the main text (PDF)
