# Supplementary material for: Ultrafast Optical Kerr Effect Spectroscopy Reveals the Vibrational Fingerprint of Acetate–Water Hydrogen Bonds
Source: ACS Omega. 2025 Dec 1;10(48):59840–8. doi: 10.1021/acsomega.5c09879 (PMC12772401; doi:10.1021/acsomega.5c09879)
Supplement: Supplementary file 1 [file ao5c09879_si_001.pdf]

Supplementary Information

Ultrafast Optical Kerr Effect Spectroscopy Reveals the Vibrational Fingerprint of Acetate–Water Hydrogen Bonds

Yousaf Shah,[1] Stephen R. Meech[2] and Ismael A. Heisler[1,*]

[1]*Instituto de Física, Universidade Federal do Rio Grande do Sul - UFRGS, Avenida Bento Gonçalves, 9500, Porto Alegre, Brazil*

[2]*School of Chemistry, Norwich Research Park, University of East Anglia, Norwich NR4 7TJ, UK*

[*]Corresponding author: ismael.heisler@ufrgs.br

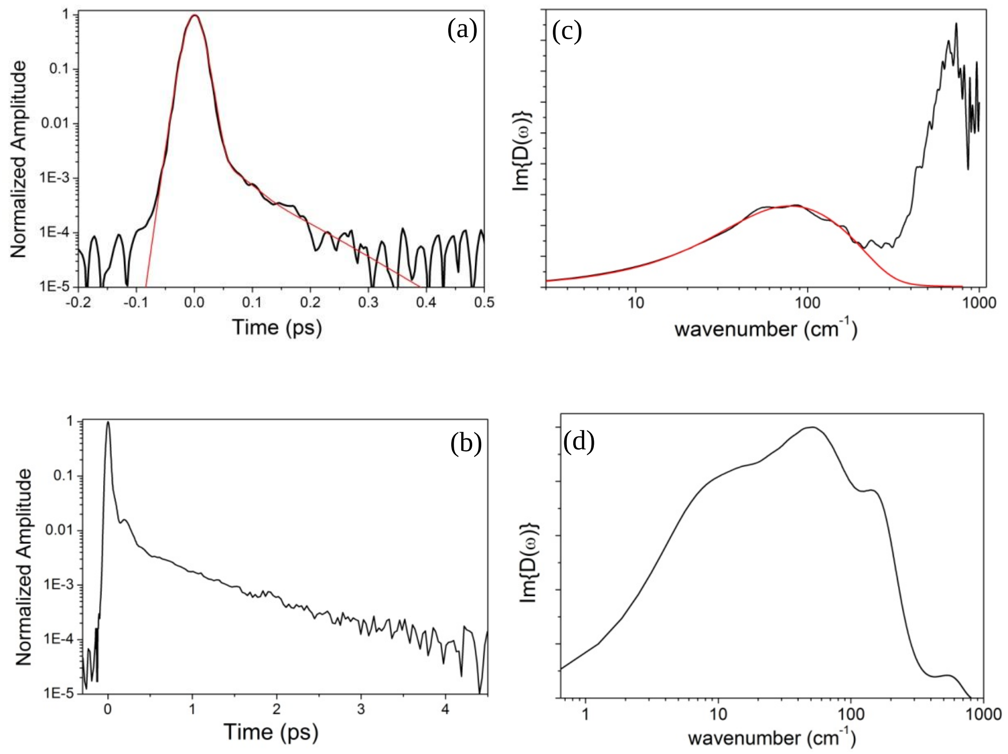

Figure S1. Pure water data (black) and fit (red) for (a) $R_{\mathrm{ISO}}(t)$ response and (b) $R_{\mathrm{ANISO}}(t)$ response. Frequency domain data are shown in (c) $R_{\mathrm{ISO}}(\omega)$ response and (d) $R_{\mathrm{ANISO}}(\omega)$ response. The spectral density (vertical axis) is obtained from the imaginary part of the Fourier transform of the OKE signal divided by the measured autocorrelation at the sample position.

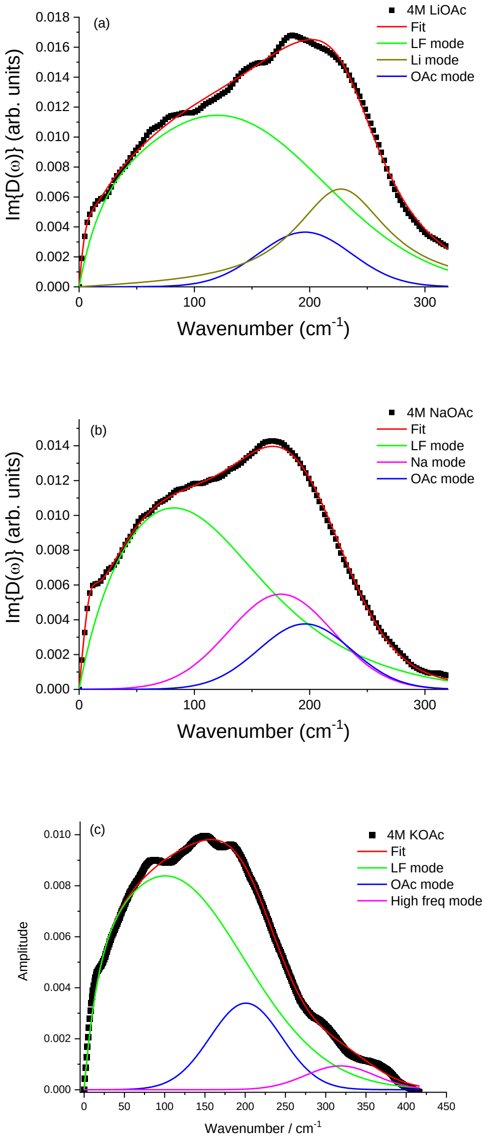

Figure S2. Isotropic OKE spectra of acetate salts with different cations, (a) Li^+, (b) Na^+ and (c) K^+. In all cases, a vibrational band appears at ~200 cm^{-1} with negligible frequency shift, demonstrating that the feature arises from acetate–water hydrogen bonds and is insensitive to the identity of the counter-cation.

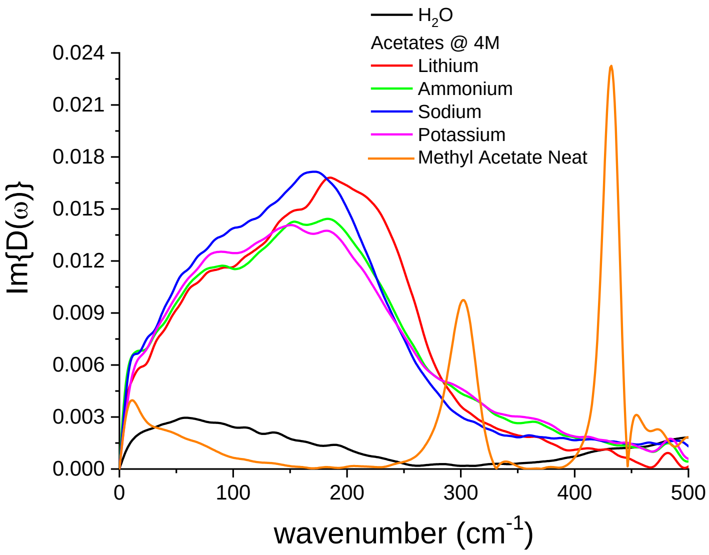

Figure S3. Isotropic OKE spectra of acetate solutions at 4 M with different cations (Li^+, Na^+, K^+, NH_4^+), compared with neat water and methyl acetate. The ~200 cm^{-1} acetate–water band remains invariant across cations, confirming its origin in the carboxylate–water hydrogen-bond interaction.

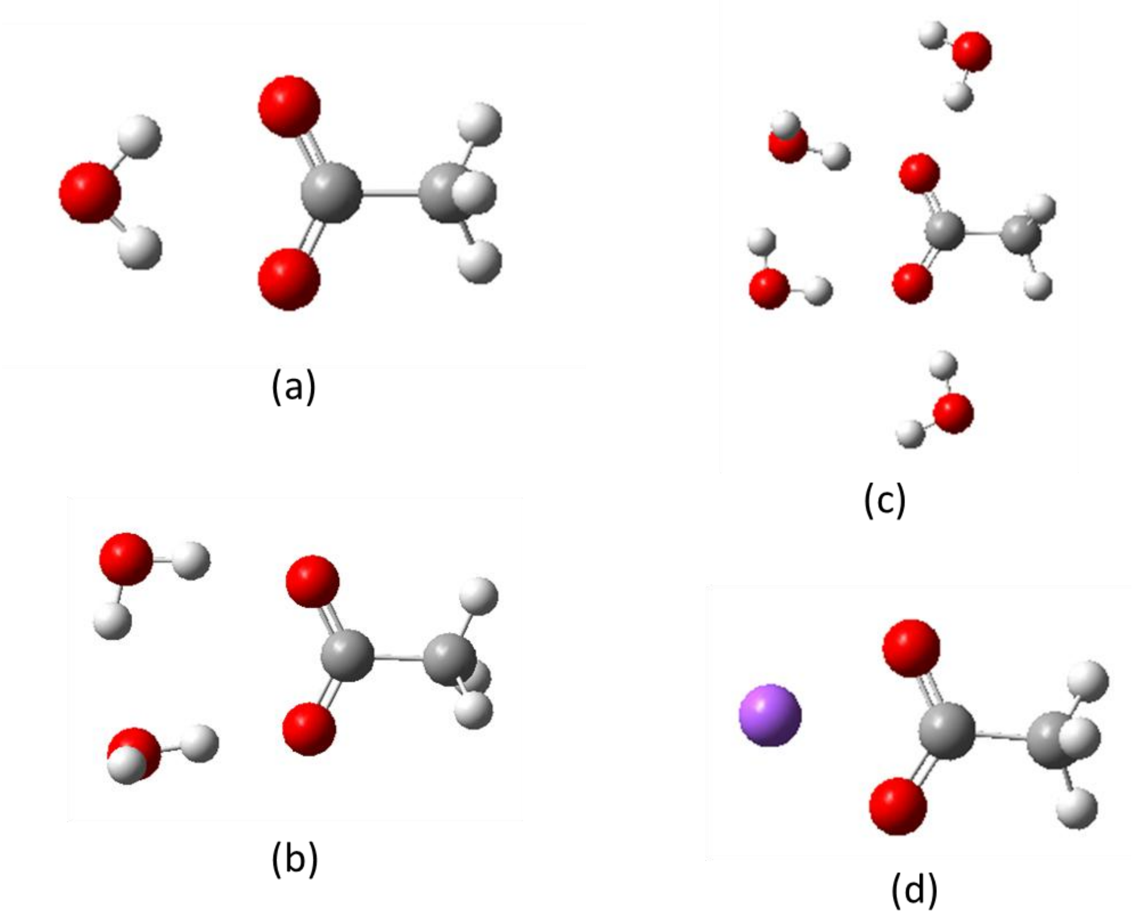

Figure S4. DFT optimized structures. (a) Acetate ion plus one water molecule. (b) Acetate ion plus two water molecules. (c) Acetate ion plus four water molecules. (d) Acetate ion plus sodium cation.

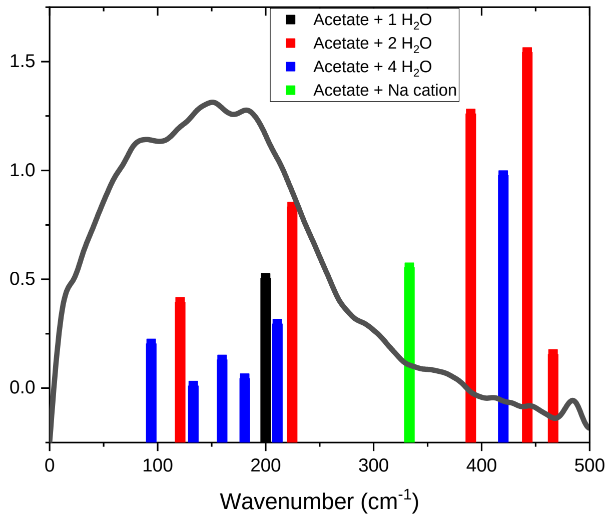

Figure S5. Experimental isotropic OKE spectrum of 4 M KOAc (gray) overlaid with DFT stick spectra for acetate–$(H_2O)_n$ clusters and the Na^+–acetate ion pair. Bars represent harmonic vibrational modes scaled by relative Raman intensity: black = acetate + 1 H_2O, red = acetate + 2 H_2O, blue = acetate + 4 H_2O, and green = acetate + Na^+. The overlap near 200 cm^{-1} highlights the polarized acetate–water hydrogen-bond stretch that dominates the isotropic OKE response. Only modes with depolarization ratios < 0.5 are shown.

Table SI. DFT calculated normal-mode harmonic frequencies, the Raman activity, and the depolarization ratio calculated for optimized structures as shown in Figure S4.

(a) Acetate anion plus one water molecule

Nº	Freq (cm^{-1})	Raman	Depolar-P
1	35.6792	0.8742	0.75
2	54.3249	1.154	0.7469
3	86.0409	0.1009	0.7491
4	200.016	0.5058	0.1308
5	287.83	0.9884	0.7499
6	334.717	0.0945	0.75
7	448.309	0.0769	0.7499
8	575.503	3.8593	0.7412
9	618.702	2.4734	0.4739
10	644.087	0.4297	0.5261
11	888.037	32.9051	0.1625
12	1005.93	0.2375	0.7495

(b) Acetate anion plus two water molecules

Nº	Freq (cm^{-1})	Raman	Depolar-P
1	30.4595	1.0024	0.7485
2	39.9731	0.4937	0.7094
3	58.2127	0.6555	0.747
4	88.8975	0.3357	0.6453
5	120.881	0.3959	0.4205
6	136.743	0.0178	0.75
7	185.379	0.059	0.7295
8	224.513	0.8338	0.1833
9	335.227	1.1043	0.6986
10	389.896	1.2613	0.3098
11	442.262	1.5439	0.3021
12	466.117	0.1564	0.5314
13	571.009	0.1532	0.75
14	617.039	1.2837	0.4853
15	660.171	0.6868	0.4947
16	705.725	0.4164	0.4727
17	925.893	30.1772	0.1665
18	1001.61	0.6177	0.7447

(c) Acetate anion plus four water molecules

Nº	Freq (cm^{-1})	Raman	Depolar-P
1	9.3966	0.3906	0.712
2	24.5382	0.0393	0.732
3	26.3947	0.0431	0.734
4	28.8793	0.0568	0.715
5	33.7906	0.0683	0.733
6	47.6477	0.6512	0.719
7	63.4019	0.5676	0.746
8	82.1848	0.2186	0.737
9	94.0847	0.2052	0.2468
10	100.058	0.7608	0.7147
11	133.009	0.0116	0.2238
12	150.548	0.2106	0.6173
13	159.744	0.1317	0.1325
14	180.638	0.0453	0.4843
15	210.891	0.2962	0.1484
16	231.589	0.2931	0.743
17	312.954	0.9865	0.6027
18	318.479	0.5407	0.7218
19	326.028	0.6713	0.7136
20	373.1	0.7501	0.7068
21	420.003	0.9784	0.5137
22	475.888	0.0527	0.6652
23	528.388	1.1706	0.7348
24	569.723	1.0901	0.6245
25	589.76	0.341	0.7744
26	620.706	0.8289	0.6177
27	633.631	2.1693	0.6347
28	727.094	0.6665	0.7611
29	927.903	0.9953	0.7303

(d) Acetate anion plus sodium cation

Nº	Freq (cm^{-1})	Raman	Depolar-P
1	22.0337	0.6161	0.75
2	111.165	0.5224	0.7484
3	240.668	0.0082	0.75
4	333.099	5.4961	0.207
5	462.604	0.0573	0.75
6	621.219	1.1922	0.6959
7	677.479	0.8833	0.1327
8	930.459	28.7657	0.0981
9	1024.9	1.0695	0.75
10	1065.45	0.4079	0.5991
11	1367.85	2.661	0.4339
12	1427	41.053	0.51
13	1462.93	10.0713	0.75
14	1483.16	8.7258	0.623
15	1570.4	3.4243	0.75
